# High intensity of *Tunga penetrans* infection causing severe disease among pigs in Busoga, South Eastern Uganda

**DOI:** 10.1186/s12917-017-1127-z

**Published:** 2017-06-29

**Authors:** Francis Mutebi, Jürgen Krücken, Hermann Feldmeier, Charles Waiswa, Norbert Mencke, Wilfred Eneku, Georg von Samson-Himmelstjerna

**Affiliations:** 1 School of Veterinary Medicine and Animal Resources, College of Veterinary Medicine, Animal Resources and Biosecurity, Makerere University, P.O. box 7062 Kampala, Uganda; 2 Institute for Parasitology and Tropical Veterinary Medicine, Freie Universität Berlin, 14163 Berlin, Germany; 3 Institute of Microbiology and Hygiene, Charité University Medicine, Berlin Campus Benjamin Franklin, Sylter Straße 2, 13353 Berlin, Germany; 4 Bayer Animal Health, 51368 Leverkusen, Germany

**Keywords:** *Tunga penetrans*, Pigs, Severe, Tungiasis, Uganda

## Abstract

**Background:**

Towards the improvement of stakeholders’ awareness of animal tungiasis, we report 10 unusual severe clinical cases of pig tungiasis which were associated with very high infection intensities of *T. penetrans* in an endemic area.

**Results:**

Morbidity of ten pigs with high sand flea intensities detected during high transmission seasons in an endemic area in Busoga sub region, Uganda is described in detail. The cases of pigs presented with a very high number of embedded sand fleas (median = 276, range = 141–838). Acute manifestations due to severe tungiasis included ulcerations (*n* = 10), abscess formation (*n* = 6) and lameness (*n* = 9). Chronic morphopathological presentations were overgrowth of claws (*n* = 5), lateral deviation of dew claws (*n* = 6), detachment (*n* = 5) or loss of dew claws (*n* = 1). Treatment of severe cases with a topical insecticidal aerosol containing chlorfenvinphos, dichlorvos and gentian violet resolved acute morbidity and facilitated healing by re-epithelialisation.

**Conclusions:**

The presentations of tungiasis highlighted in this report show that high intensities of embedded *T. penetrans* can cause a severe clinical disease in pigs. Effective tungiasis preventive measures and early diagnosis for treatment could be crucial to minimize its effects on animal health.

## Background

Tungiasis, a zoonotic parasitic dermatosis of humans and a wide range of domestic and wild mammals, is caused by the female penetrating sand flea, *Tunga penetrans*. Currently, it is endemic in Latin America, the Caribbean and sub-Saharan Africa with a patchy distribution; mostly occurring in poor communities and often causing severe morbidity in both human and animals [1]. The disease is largely neglected in tropical human and veterinary medicine [2]. Pigs have been identified as the most important animal reservoirs of *T. penetrans* in sub-Saharan Africa. Frequently, they suffer from high parasite loads and severe morbidity [3, 4]. Most sand fleas localize on the coronary band and bulbs of the digits [5–7] but other body parts which contact or are close to the ground such as mammary glands, snout, legs, perineum and the tail may also be affected [1].

Proper and early diagnosis of tungiasis is essential for timely and appropriate treatment as well as control in order to abate its effects on animal health. Studies focusing on the clinical presentations of tungiasis and its significance to animal wellbeing and health are very limited. Consequently, many animal health workers, at least in the highly endemic areas in Uganda, are not aware of the clinical significance of tungiasis and often consider it an insignificant nuisance which hardly requires veterinary medical care (Mutebi, unpublished findings). Hence, many cases go unattended despite detrimental effects on public and animal health. The accruing losses may hamper economic development of impoverished communities located in endemic areas.

Clinical tungiasis in pigs has barely been described. We have recently described and analyzed the general manifestations of tungiasis in pigs [5]. The study identified a wide range of associated clinical presentations such as hoof wall erosion, necrosis, pain, edema and skin fissures in a population of pigs with 1–246 lesions per animal (median 8) [5]. The aim of this case report series is to describe in detail severe pig tungiasis detected during the same survey and a later treatment study on animal tungiasis in Busoga sub region, Uganda, which were conducted during the dry seasons of January to March in 2014 and 2015 [4, 8]. The findings indicate that during periods of high transmission, pigs may harbor very high intensities of embedded sand fleas leading to severe clinical and pathological manifestations.

## Methods

### Study villages and pig population

Severely infected pigs were identified in two endemic villages (Busindha and Masolya, Bulidha Sub County, Bugiri District) during a survey and a treatment study [4, 8]. Since the intensity of embedded sand fleas strongly correlates with the severity of clinical tungiasis [9], only pigs with over 100 embedded sand fleas are presented here to demonstrate the occurrence and clinical features of severe pig tungiasis caused by *T. penetrans*. All pigs were of mixed breeds (unknown parentage due to random mating) whose management system was rather homogeneous in the two villages. In all cases, pigs had no formal housing but were occasionally confined under tree/shrub shades close to human compounds (0–10 m) with minimal attention to hygiene. Thus, the grounds of the pig dwellings were heavily contaminated with wasted pig feeds and feces. During the crop harvesting periods, which are the dry seasons, the pigs were allowed to roam on compounds and the neighborhood with minimal restrictions. Parasite control was not routinely undertaken and farmer animal husbandry advisory services were generally lacking. Pigs and goats were the major livestock but other domestic mammalian and avian species were also being raised.

### Diagnosis of tungiasis

A systematic clinical examination was performed after thorough washing of the digits with soap, water and a scrubbing brush to aid lesion detection. Diagnosis of tungiasis was based on clinical features of tungiasis which included: a dark brown to black spot surrounded by a zone of hyperemia or edema (stage II), a 2–13 mm circular raised yellow to white nodule with a dark center (stage III), a circular and raised brown to black patch in the middle of a necrotic area with or without erosions or ulcers (stage IV) and an epidermal circular shallow crater with necrotic edges (stage V) [10]. In a detailed clinical examination, lesions were assigned to a particular stage (I-V) according to the Fortaleza classification [10] as briefly described above, counted and their location noted as infection sites. Since most lesions occurred on the distal limb, each limb was divided into four topographic sites each representing a principal or an accessory digit up to the distal metacarpal or metatarsal joints. This results in 16 digits for the four limbs of a pig. Embedded sand fleas which were localized on other additional sites of the body such as the scrotum, tail, snout and the skin along the metacarpals or metatarsals were also recorded and considered ectopic. The clinical and pathological features which were associated with the embedded sand fleas were also described in detail and reported. The penetrated fleas were identified as *T. penetrans* [4] based on the morphological features of the neosomes [1, 11]. The 10 pig cases selected and described here were those with the highest number of embedded sand fleas among a total of 183 infected pigs identified during the survey [4] and the field treatment trial [8].

### Treatment of pigs with embedded sand fleas

All cases were treated with the Supona® aerosol (chlorfenvinphos, dichlorvos and gentian violet; previously Pfizer Laboratories (Pty) Ltd., now Zoetis, South Africa), a formulation licensed in Uganda to treat tick infestations, myiasis and wound sepsis in animals. The aerosol was applied on all the 16 digits of affected pigs after washing them with water and a brush. The aerosol was only applied to other ectopic sites when they had embedded sand fleas. Pigs were treated weekly for three consecutive times.

### Statistical analysis

Data were entered into Microsoft Excel 2007 sheets, double checked against written data collection forms and then transferred to Stata® Software package, Version 13 (Stata Corporation, College Station, Texas 77,845 USA, stata@stata.com) for analysis. Descriptive statistics were generated.

## Results

### General demographic, management, parasitological and clinical characteristics of the ten pigs with severe tungiasis

The pigs came from two villages (Masolya and Busindha) with a high prevalence of human and animal tungiasis and were identified in five households in which at least one human being was also infected by tungiasis. Cases 1–3 were detected during the epidemiological survey (January to March, 2014) while the remaining pigs were identified in the following year (January to March, 2015). While four pigs came from households that had only a single pig, the other six pigs (cases 4–9) were identified from a single household in Masolya. The six infected pigs which were identified from the same household presented with the highest number of lesions. The clinical presentations of each of the ten pigs are described in detail and the demographic and management information is summarized in Table 1.

**Table 1:** Demographic and management characteristics of ten pigs with severe tungiasis

Parameter	Proportions or value
Village (number of pigs/total number of 10 pigs)	
Masolya	9/10
Busindha	1/10
Age in months: Median (range)	8.3 (2.5–24)
Sex ratio (Male/Female)	3/7
Management system Scavenging/tethering^a^ (number of pigs/total number)	10/10
Ectoparasite control: (Number of pigs/total number)	0/10
Helminth control^b^ (number of pigs/total of 10 pigs)	1/10

^a^Management changed during the seasons of the year

^b^Used 150 mg tablet of levamisole (Wormicid® 150, Cosmos, Kenya)

Due to the high parasite load, some lesions were not discrete and hence only those which were distinct were documented. A total of 3834 lesions were counted among the 10 pigs of which 1676 (43.7%) were viable (stage IIa-IIIb) while 2158 (56.3%) were dead (stage IV-V) of which the majority were excoriated. The median number of lesions per pig was 276 and the range was 141–838 lesions. Sand fleas were embedded on legs in all the10 pigs, scrotum (*n* = 2), snout (*n* = 2) and the tail (*n* = 1). Eight of the ten pigs had lesions on all the four legs while for the other two pigs, sand fleas were detected on three legs. The number of discrete sites with embedded sand fleas per pig ranged from 9 to 20 with a median of 17.5 sites per pig. A median of eight principal digits (range = 5–8) were affected while the number of accessory digits with sand fleas ranged from three to eight (median = 7.5) per pig. Most lesions were clustered at the infected sites resulting in an appearance reminiscent of a honey comb especially at sites with manipulated lesions. The number of lesions detected and their location are summarized in Table 2.

**Table 2:** Localizations and number of embedded sand fleas among the ten pigs

Topographic site	Number of lesions	
	Median (range)	Total (% of all lesions, *n* = 3834)
All legs	272 (141–744)	3672 (95.8%)
Front legs	123 (4–366)	1515 (39.5%)
Hind legs	157 (112–379)	2157 (56.3%)
All digits	142 (25–399)	3322 (86.6%)
Principal digits	168.5 (92–399)	2150 (56.1%)
Accessory digits	97 (25–250)	1172 (30.6%)
Metacarpal/metatarsal skin	8.5 (0–104)	350 (9.1%)
Sites other than legs (ectopic)	0 (0–94)	162 (4.2%)
Scrotum	0 (0–94)	106 (2.8%)
Snout	0 (0–19)	31 (0.8%)
Tail	0 (0–25)	25 (0.65%)

Despite a high parasite load, pale mucous membranes were observed in only one pig suggesting that it could be anemic and none of the affected pigs was pyrexic (rectal temperature of 38.4–39.3 °C) to implicate septicemia. Nevertheless, seven of the pigs had signs of bacterial super infections (scabs and purulent lesions).

All pigs had abraded lesions and ulcers at sites of sand flea penetration, a feature which suggests pruritic lesions among all the pigs. Three pigs were observed intensely rubbing affected sites on the ground. All affected sites were painful among pigs as evidenced by jerking after applying modest digital pressure. All the ten pigs had both acute and chronic manifestations of tungiasis observed at various affected body sites of the pigs.

When asked about the health status of their pigs, the three pig owners whose pigs exhibited some degree of lameness were able to report it but none of them had an idea of what its cause could be. Also none of the remaining pig farmers with severely infected pigs knew that their pigs were infected by *T. penetrans*. Moreover, none of the two animal health workers working in the study district who were interviewed had ever encountered any case of animal tungiasis. The clinical presentations of tungiasis among the severely affected pigs are summarized in Table 3.

**Table 3:** Tungiasis-associated clinical presentations among the ten severely affected pigs

Clinical feature^a^	Number affected/number of examined animals
Congestion	10/10
Edema	10/10
Ulcers and erosions	10/10
Fissures	10/10
Necrosis at affected sites	10/10
Abscessation/purulent lesions/scabs	6/10
Lameness	9/10
Hyperkeratosis	3/10
Deformations	8/10
Lateral deviation of dew claws	6/10
Overgrowth of hooves	5/10
Detaching/loose hooves	5/10
Loss of dew claws	1/10

^a^One or more affected topographic sites involved

### Case 1

**Fig. 1:**
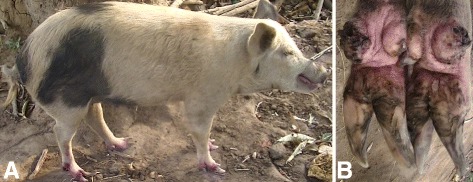
Severe chronic tungiasis in a pig (case 1). **a** Heavily infected pig with feet showing violet colour from treatment with Supona®. **b** There is bilateral loss of the lateral dew claws on the hind legs in the sow while the remaining median dew claws are deviated laterally and deformed while the hooves are overgrown

A one year old sow was found in a household where all humans were infected with *T. penetrans*. It had a total of 246 lesions of which 202 were viable sand fleas (stage II and III) while 44 had degenerated lesions (stage IV and V) according to the Fortaleza classification [10]. The lesions were distributed on 15 of the 16 digits of the four legs as well as the skin along the metatarsal and metacarpal of one hind and front leg. It had completely lost three dew claws, three of the persisting dew claws were only represented by thin rudimental and deformed vestiges while all the remaining dew claws and the principal digital claws were laterally deviated and overgrown (Fig. 1). The sites of sand flea localization were painful on palpation and edematous while a few were hyperemic. The pig exhibited alteration of gait during movement. It was also heavily infested with lice.

### Case 2

**Fig. 2:**
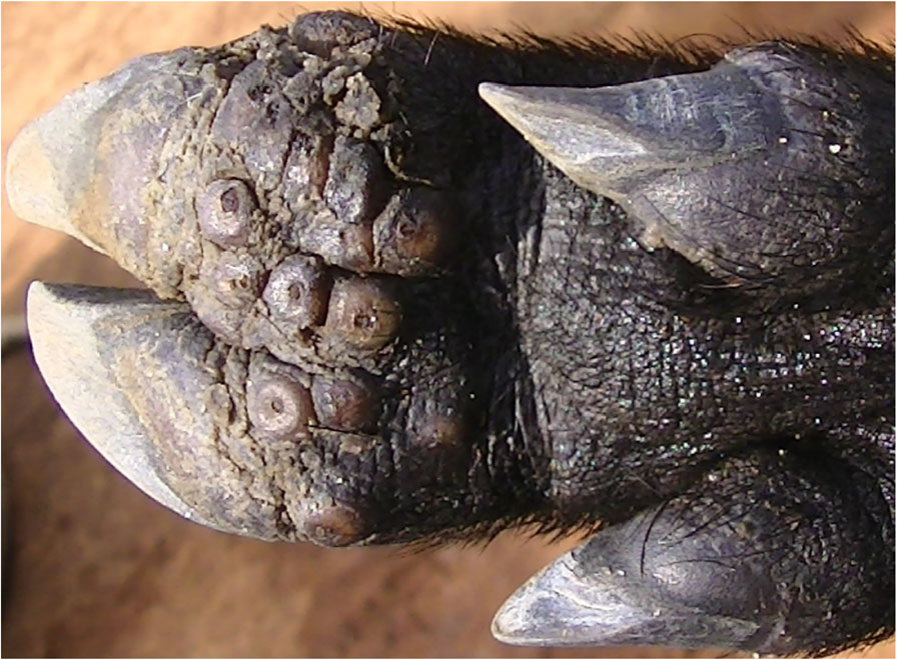
Digits of a hind limb of a sow with severe tungiasis (case 2). The digits have cracks between the embedded sand fleas and the intervening tissues are extensively necrotic

An eight month old pregnant sow was found to harbor a total of 170 lesions of which 112 were viable and 58 non-viable. All lesions were confined to the legs on the main and accessory digits. There were extensive ulcerations and necrosis of the skin and the hoof wall of the affected digits (Fig. 2). The affected accessory digits were swollen; all principal digits had fissures, exhibited hyperkeratosis and desquamation. Despite a high number of embedded sand fleas, no functional limb use alterations were detected but it was observed repeatedly rubbing the affected digits against the ground.

### Case 3

**Fig. 3:**
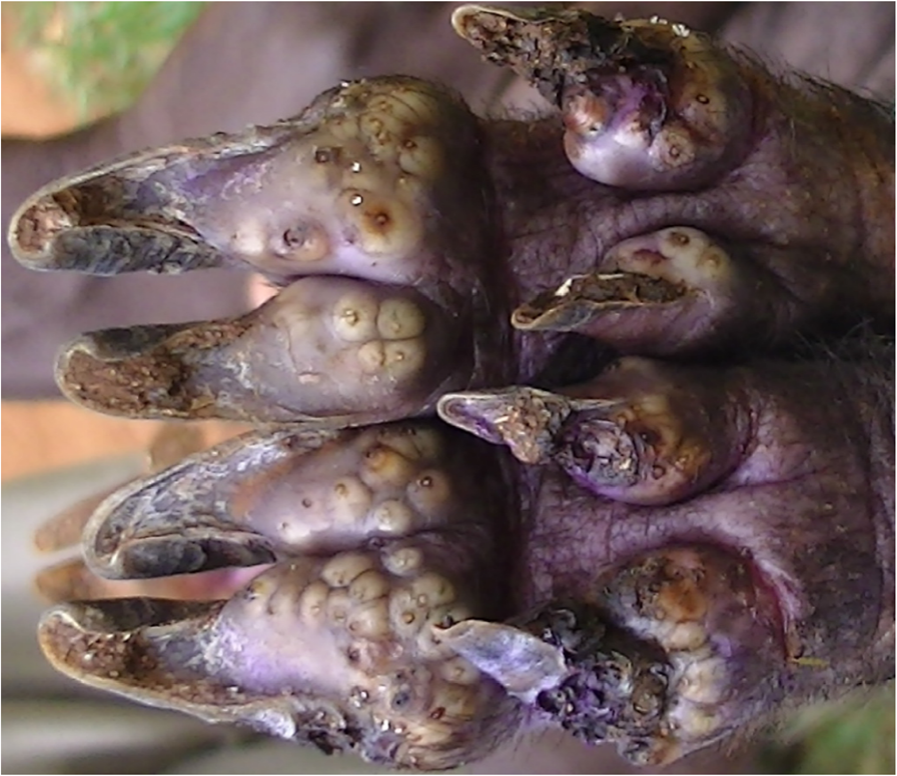
Two hind limbs of a pig with numerous sand fleas on all the digits (case 3). Extensive necrosis of the hoof walls is visible at sites of sand flea penetration and the lateral dew claw is only loosely attached

A five month old female piglet had 141 lesions in total of which 107 were viable and the rest dead. All lesions were on the legs, only two were localized on the right front leg while the remaining lesions were on the hind legs. The claws of four digits were loose and almost falling off while the remaining hooves of the hind legs were overgrown (Fig. 3). The skin and hoof wall of the affected sites were extensively necrotic and ulcerated. The sites of sand flea clustering were swollen and painful on palpation. It was recumbent most of the time, had difficulties in movement and was grossly anemic. It was seen intermittently rubbing the affected hind legs on the ground. Generally, it was emaciated and heavily infested by lice and had mange. The owner reported to have dewormed it with levamisole (Wormicid® tablet, cosmos limited, Kenya) a month before it was examined.

### Case 4

**Fig. 4:**
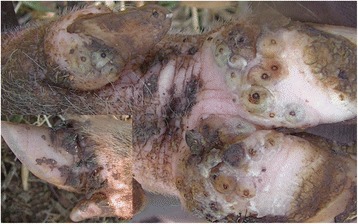
Digital sand flea lesions in a pig (case 4). The inset shows fissures along the coronary band on the hind lateral digit

This was a two year old lactating sow with eight piglets. It had 297 lesions of which 208 were viable and the rest dead and it was generally emaciated. All lesions were on legs on the digits as well as the skin along the metacarpals and metatarsals where there were injuries of ropes used for restraining the pig. The eight main digits were ulcerated and had fissures at the coronary bands (Fig. 4). There was extensive hoof wall erosion, skin necrosis and scab formation at sites of sand flea localization. All affected sites had a foul smell. The areas with lesions were hyperemic and affected digits were swollen and painful which was reflected by movement difficulties. The hooves of three dew digits were detached at their respective cranial aspects of the digit. The lateral dew claw of the left front leg was laterally deviated and overgrown. The pig also had lice and was infested by ticks.

### Case 5

**Fig. 5:**
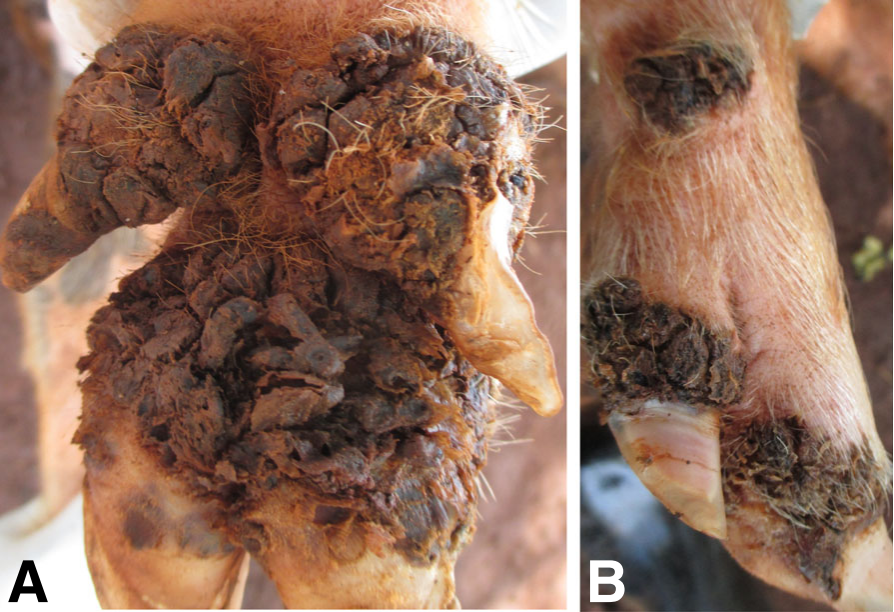
High number of embedded *T. penetrans* sand fleas on pig digits (caudal view **a** and lateral view **b** (case 5). There is marked excoriation of lesions giving an impression of a honeycomb (**a** and **b**) The dew claws were also laterally deviated **a**

A boar of six months was identified with 136 viable lesions and over 838 lesions in total. Ninety-four of the lesions were on the scrotum while the rest were on the digits around the coronary band and the hoof bulb as well as the skin along the metacarpal and metatarsal bones. The majority of the lesions was mutilated by intense rubbing against the ground and objects hence most lesions were non-viable and the affected sites were necrotic and ulcerated. Because of clustering of the lesions on the affected sites, the lesions were reminiscent of a honeycomb (Fig. 5a). The scrotum and the affected digits had multifocal small abscesses and/or scabs and had a foul smell. The digits were swollen and the junction between the necrotic affected sites and the adjacent skin were hyperemic (Fig. 5b). The pig moved with difficulties and was reported to be recumbent most of the time. Three of the accessory digits of hind legs were deviated laterally. It also had tick and lice infestations.

### Case 6

**Fig. 6:**
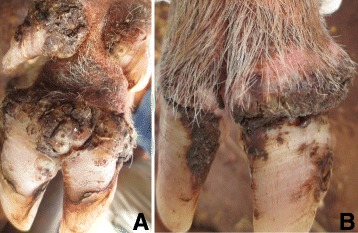
Distal hind legs (caudal view **a** and cranial view **b**) (case 6). Affected sites are swollen, cracked and necrotic while the surrounding area appears congested **b**

A one year old boar had 188 viable lesions and over 398 lesions in total. Of the embedded sand fleas, 12 were localized on the scrotum while the rest were on the legs at the coronary band and hoof bulb as well as the adjacent skin on the metacarpal and metatarsal bones at sites of ulcerations or abrasions by the restraining ropes. The affected digits were swollen and sites of sand flea localization were diffusely hyperemic and necrotic (Fig. 6a & b) while the skin-hoof junction had fissures at various sites. Affected digits were painful with minimum digital pressure and scabs were evident at various points on affected sites. The lesions had a foul smell and the hoof wall surface was eroded. The pig exhibited supporting lameness of the hind legs while moving. It was also infested by ticks and lice.

### Case 7

**Fig. 7:**
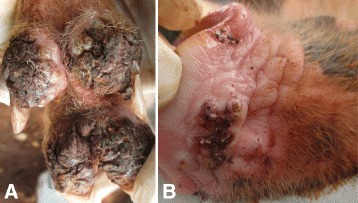
Distal hind limb and snout of a piglet (case 7). Affected sites on the digits are swollen, necrotic and cracked **a**. The snout was also affected and showed mutilated lesions and ulceration **b**

This case was a male piglet of three months of age whose sow and other seven littermates were also affected by tungiasis, though milder. It had 126 viable embedded sand fleas and over 255 lesions in total that were distributed over 17 different sites which included all the 16 limb digits (243) and the snout which had 12 lesions. The majority of the lesions were mutilated by intense rubbing of the affected sites against the ground and other objects. All affected sites were extensively necrotic, ulcerated, swollen and scabby with fissures (7A). Hyperemia was evident at the junction between the sites where lesions were clustering and the adjacent normal area. The lesions had a bad smell and on most digits the coalescent clusters of sand fleas appeared like a honeycomb. Two dew claws were loosely attached and another two were growing laterally away from the normal plane. All sites were painful on touch and the piglet limped while moving. The lesions on the snout were also mutilated resulting in snout ulceration and hyperemia (Fig. 7b). The piglet was also heavily infested by ticks and lice.

### Case 8

**Fig. 8:**
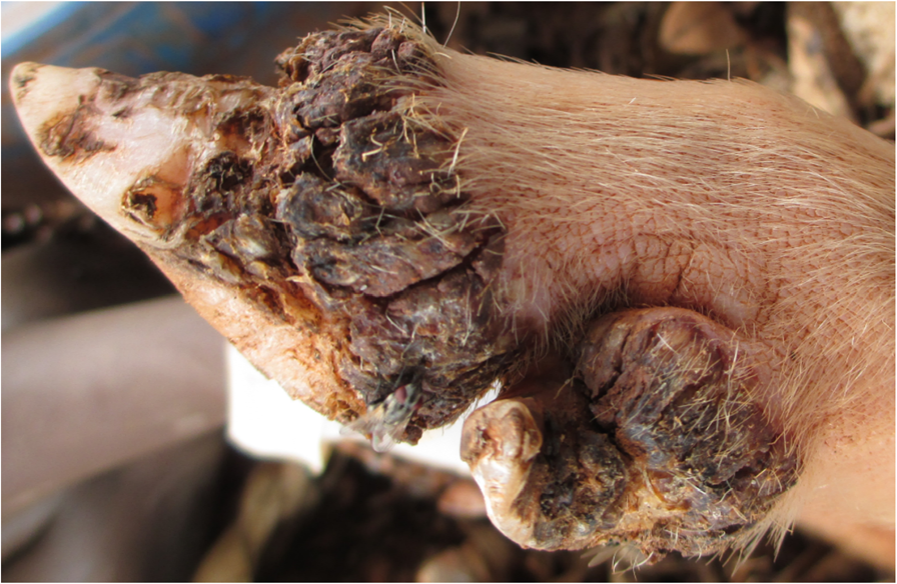
Hind leg digits of pig with extensive necrosis, abscessation, ulcerations and cracks (case 8). The lateral dew claw is deformed with lateral deviation and pronounced hyperemia at the edge of the affected site

This was a six month old female pig that had 288 viable embedded sand fleas and over 694 *T. penetrans* lesions in total. These were distributed on 19 sites on the legs which included all the 16 limb digits as well as the skin along the two metacarpals and the left hind leg metatarsal which were traumatized by a restraining rope. Affected sites were painful and swollen with coalescing ulcers, fissures, extensive necrosis, scabs or abscesses and swollen. Rims of hyperemia were evident between affected sites and the adjacent normal skin (Fig. 8). The lesions had a repulsive odor. Four dew claws were laterally deviated and one was only loosely attached to the digit. The pig moved with difficulty and was reportedly recumbent most of the time. Lice and tick infestation were also apparent.

### Case 9

A female pig of six months had 226 viable lesions and over 614 lesions in total. Sand fleas were embedded on over 18 sites, which included all the sixteen digits as well as the snout and the tail. Of the lesions, 19 were on the snout while 25 were on the tail and the rest on the digits. All affected sites were ulcerated, swollen, very painful when applying minimal digital pressure and extensively necrotic. The digits and the tail also had fissures. In addition, hyperemia was evident at the edges of the affected sites and scabs were present on all digits with isolated pockets of suppuration on digits and the tail. The tail was kinked at the site of sand flea attachment. One dew claw was almost detached and two accessory digits were growing laterally. Digital lesions associated with sand fleas had a foul smell and the pig had walking difficulties. It was also infested by lice and ticks.

### Case 10

A female piglet of three months was being reared with another piglet of the same age, which was mildly infected. The piglet had 181 lesions of which 83 were viable distributed over nine digits. Affected digits were diffusely ulcerated, necrotic and intensely hyperemic. Fissures were also evident at the coronary band and between hypertrophic sand fleas at sites of heavy infection. Despite a high number of sand fleas, no functional limb disturbances were evident. The piglet also suffered from lice infestation.

### Outcome of treatment of cases

**Fig. 9:**
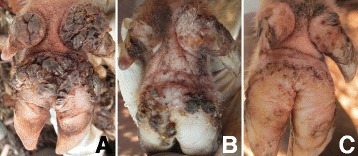
Distal hind leg of one pig before treatment **a**, one week after first treatment **b** and one week after second treatment **c** (case 9). It had 226 viable lesions before treatment, seven after one treatment and none after two treatments

**Fig. 10:**
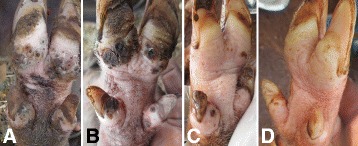
Distal hind leg of a pig before treatment **a**, one week after the first treatment **b**, a week after the second treatment **c** and one week after the third treatment **d** (case 4). It had a total of 208 lesions before treatment, three lesions one week after the first treatment and none a week after the second treatment and the third treatment

Following treatment, the lesions cleared by the second or third week and healing by re-epitheliazation occurred (Figs. 9 and 10). The acute morbidity signs and lameness associated with the infection were abated. However, the chronic features of tungiasis such as digital deformities and lameness caused by detaching of hooves persisted throughout the treatment and observation period.

## Discussion

Despite the severe morbidity due to *T. penetrans* infections in animals [1, 5, 7], tungiasis remains among the under diagnosed, under reported and hence neglected diseases of animals [2]. With the exception of a single report of tungiasis-associated agalactia in sows [12], tungiasis in pigs has been mainly depicted as a disease with minimal effects on animal health [6, 13, 14]. Recently, the major manifestations of tungiasis in pigs and dogs, some of which with significant effects to animal health were described [5]. Unfortunately, tungiasis mainly occurs in impoverished communities with limited veterinary services. Also there is limited awareness of animal health workers on the impact of tungiasis on animal health. These circumstances may contribute to the wrong impression that tungiasis is a mere nuisance to animals. Consequently, many cases go undetected and are therefore not treated [1].

The ten clinical cases described had more than 100 lesions per pig. This data shows that high intensity of infection causes a severe morbidity, a finding very similar to the human situation [15]. This further highlights the animal health significance of *T. penetrans* infections in pigs. To the best of our knowledge, the parasite loads reported here in some pigs constitute the highest intensity of *T. penetrans* ever reported among pigs or any other susceptible mammalian species [1, 11, 14, 16].

There was an extremely high number of sand fleas per individual animal of which most of the lesions were condensed on a limited skin surface area. Whether this observation represents a high infection pressure from the environment or even an aggregation by the sand fleas themselves needs to be subjected to further investigations.

Once a sand flea penetrates the skin, it increases in size, evoking severe inflammation and itching [10]. The later predisposes the host to self-mutilation through scratching of affected sites or rubbing them against other objects or hard surfaces. The resulting erosions, ulcers, necrosis and skin cracks create entry points of pathogenic bacteria and facilitate penetration by free living sand fleas. This, together with the lack of regular on-host ectoparasite control among the pig owners, may have contributed to the high infection intensities and severe morbidity observed among the pigs.

A high rate of bacterial super infections through sand flea infected parts of the skin among pigs with high sand flea burden probably contributed to the significant clinical and morpho-pathological findings. Furthermore, repeated infections and the associated sequelae contributed to the deformities, loss of claws and lameness as seen in the cases presented here. Lameness accruing from painful lesions on the digits may limit pig movement. Considering the scavenging management system in the study area, reduced pig movement is likely to lower the pigs’ ability to search for food, an outcome which may contribute to stunted growth. Also, tungiasis may contribute to reduced market value of affected pigs. Persistence of animal tungiasis in endemic communities may deter their economic development. In order to arouse more interest of stakeholders on animal tungiasis, studies appraising the economic significance of pig tungiasis should be carried out in endemic areas.

An ancillary finding is the observation that 9/10 severely infected pigs were co-infested with at least one other ectoparasite (ticks, mites and/or lice). Again, the situation is very similar to that in human tungiasis, where affected individuals showed co-infection with *Sarcoptes scabiei*, head lice or zoonotic hookworm larvae migrating in the skin [17].

Fortunately, diagnosis of tungiasis is easy and cheap since it is based on visual detection of characteristic *T. penetrans* lesions in the skin usually of the digits. Early detection and treatment are critical for quick recovery. After treatment with an insecticidal aerosol, healing occurred with re-epithelialization of the skin in uncomplicated cases [8]. However, severe cases require two or three weekly applications as demonstrated here. Nevertheless, this treatment protocol enables farmers in endemic areas to manage any cases as soon as they are detected. This has the potential to prevent tungiasis-induced lameness and other complications in the absence of veterinary services. Furthermore, such a treatment schedule is expected to reduce the contamination of the environment with *T. penetrans* eggs and thereby contribute to improved human health in endemic communities. Besides the treatment of clinical cases, efforts should also be geared towards farmer education on the diagnosis, prevention and control of tungiasis among animals as well as humans.

## Conclusion

The report demonstrates very high numbers of embedded sand fleas that were associated with severe tungiasis among free ranging pigs in a tungiasis-endemic area, which is the opposite of what local farmers and animal health workers expected. Therefore, improved pig tungiasis control practices are mandatory for improved pig health in highly endemic regions. Studies on the economic significance of animal tungiasis ought to be undertaken in order to stimulate interest in the disease among various stakeholders.

## Abbreviations

Ltd: Limited
n: Number
Ref: Reference
USA: United States of America
